# Surgical treatment of acute TB spondylitis: indications and outcomes

**DOI:** 10.1007/s00586-012-2455-0

**Published:** 2012-08-16

**Authors:** Kin Cheung Mak, Kenneth M. C. Cheung

**Affiliations:** Department of Orthopaedics and Traumatology, Queen Mary Hospital, University of Hong Kong, Professorial Block, 5th Floor, 102 Pokfulam Road, Hong Kong, SAR, China

**Keywords:** Tuberculosis, Spine, Spinal fusion, Debridement, Instrumentation

## Abstract

**Introduction:**

Spinal tuberculosis represents a challenging disease to treat, not because of the technical expertise or the time required to cure it, but more so because of the decisions involved to treat it. The Medical Research Council (MRC) Working Party on Tuberculosis of the Spine designed trials to help address several questions.

**Methods:**

A comprehensive literature search was performed using PubMed Medline, including English articles from 1934 to 1012, which pertain to spinal tuberculosis, with special effort in tracing the 13 MRC reports. The primary focus was on disease eradication, fusion rate, and a secondary focus on both short and long-term results in terms of disease recurrence and alignment. Additional searches were made on the use of spinal implants for infection cases.

**Results:**

After reviewing MRC and non-MRC reports, it was evident that the “Hong Kong operation”, which involved radical debridement and strut grafting the lesion, produced better short-, medium- and long-term results in such aspects as fusion rate, spinal deformity and relapse of abscess/sinus. Subsequent work by others demonstrated the importance of prevention of progressive kyphosis, therefore the need to identify risk factors for these and pre-emptive measures such as kyphosis correction, careful graft selection, and instrumentation.

**Conclusion:**

Improvement in quality of life is also accompanied by higher patient expectations. Though developing nations may lack the resources now, eventually patients will demand better functional and cosmetic results after being afflicted by this disfiguring and potentially disabling disease, and the “Hong Kong operation” represented the best outcome, provided resources were available.

## Introduction

In order to understand the surgical treatment of acute tuberculous (TB) spondylitis, one needs to understand the history of its treatment. Considerable controversy existed in the past regarding whether TB spondylitis should be treated by chemotherapy alone, and allow out-patient ambulation or require in-patient bed rest, or in combination with debridement surgery or a more radical procedure. The Medical Research Council (MRC) Working Party on Tuberculosis of the Spine, which was formed in 1963, designed the prospective multicenter clinical trials that took place in Hong Kong, Korea, Rhodesia and South Africa [1–9].

Thus, in Korea various combinations of conservative treatment were compared, while in Rhodesia conservative treatment was compared to debridement. In Hong Kong and South Africa, debridement was compared to radical debridement with strut grafting.

**Fig. 1:**
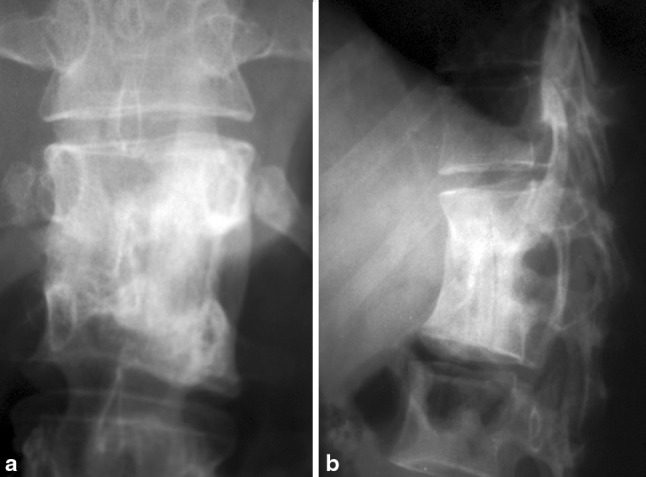
Illustration of how rib grafts are placed for the “Hong Kong Operation”, whereby the height of the rib is seen in the AP view (**a**) and the thinner profile of the rib is visible in the lateral view (**b**)

The radical surgery was first reported by Ito [10], and later popularized by Hodgson and Stock [11] after publication of their paper in 1956, and has since been called “the Hong Kong operation.” The procedure consisted of thorough excision (‘extirpation’) of the tuberculous focus, posteriorly until the dura mater, and cephalad and caudad till healthy, bleeding cancellous bone was exposed, to create surfaces suitable for docking of the strut graft. The strut graft can be from a cut rib (Fig. 1), which is obtained during the thoracotomy, or a tricortical iliac crest bone graft that offers a larger and more stable block. Resection of diseased bone may need to extend to healthy cancellous bone of the adjacent body, thus necessitating resection of the intervening disk and end plate of the healthy vertebra [3].

The Working Party laid down strict criteria for a “favorable outcome”—no symptom, full physical activity at work or school, no evidence of central nervous system involvement, no residual sinus or abscess detectable clinically or radiologically, together with radiologic evidence of *healing* of the spinal lesion [12]. Though *fusion* has never been formally proven to equate to “healing” but it has been considered “the thing aimed at” since the times of Percival Pott to this day [11]. However, there was one important omission in the definition of a good outcome. And this was the *kyphotic angle*, an important determinant not only of cosmetic well-being but also of possible future neurologic impairment.

In the earlier reviews, it was concluded that there was no significant difference in achieving “favorable outcome” when the different treatment protocols were used. In Bulawayo, Rhodesia, 83 % of patients given ambulatory chemotherapy achieved this status compared to 84 % of those with debridement surgery. The figures for Hong Kong were similar with 88 % for those debrided compared to 89 % for those with radical surgery [6], with comparable numbers from Korea and South Africa [4, 5].

Yet, if one examined the data carefully, there appeared to be clear and distinct advantages for employing the Hong Kong operation. In the short term, the procedure provided faster bony union and resolution of abscesses relative to conservative treatment, and similar results were noted when compared to debridement, as shown in Table 1, which is combining data from three MRC reports [6, 8, 12].

**Table 1:** Percentage of patients achieving bony fusion from time of presentation (data compiled from three MRC reports)

	HK rad^a^ (%)	HK deb^b^ (%)	B deb^c^ (%)	B amb^d^ (%)	K^e^ (%)
6 months	28	3	7	9	1
12 months	70	23	20	26	6
18 months	85	52	38	50	15

^a^Hong Kong center, radical debridement and strut grafting group

^b^Hong Kong center, debridement only group

^c^Bulawayo, Rhodesia center, debridement group

^d^Bulawayo, Rhodesia center, ambulatory chemotherapy group

^e^Average for Masan and Pusan in Korea, conservative treatment groups

On the other hand, once the long-term data were reviewed, an important difference in treatment outcome became clear. The Hong Kong operation not only resulted in less kyphotic deformities, the average angle of kyphosis at 5 years was less than that upon admission, as illustrated in Table 2, again with data compiled from three MRC reports [6, 8, 12]. And this alignment was maintained till the 15-year follow up.

**Table 2:** Angle of kyphosis at presentation and at 5 years (data compiled from three MRC reports)

Kyphosis (°)	HK rad^a^	HK deb^b^	B deb^c^	B amb^d^	K^e^
On admission	26	25	28	24	35
60 months	23	33	37	31	56

^a^Hong Kong center, radical debridement & strut grafting group

^b^Hong Kong center, debridement only group

^c^Bulawayo, Rhodesia center, debridement group

^d^Bulawayo, Rhodesia center, ambulatory chemotherapy group

^e^Average for Masan and Pusan in Korea, conservative treatment groups

One of the underlying principles for the anterior procedure was recognizing the importance of clearance of the paravertebral abscess in achieving fast and complete healing of TB spine [13]. Though favorable status was reached in similar proportions in the different test centers, ultimately, it was noted that Korea had relapse of sinuses, and Rhodesia had recurrence of abscesses. None of these problems were noted in the Hong Kong patients [12].

Therefore, finer analyses of the MRC trials allowed us to appreciate the advantage of radical debridement and strut grafting through an anterior approach. While the Hong Kong trial only included patients with *less than 3 vertebral bodies* involvement, the principle of requiring anterior column reconstruction is even more important in contiguous multi-level disease. Multi-level disease will be dealt with in another paper in this issue.

It is with this background that we have managed acute TB spondylitis patients surgically:

## Indications for surgery in acute tuberculous spondylitis

1. Progressive neurologic deficit
2. Progressive increase in spinal deformity (coronal or sagittal)
3. Failed conservative treatment including 1 and 2 above or severe pain due to abscess or spinal instability
4. Uncertain diagnosis: this could be an inability to obtain microbiological diagnosis from microscopy, culture or even via detection of *mycobacterium* DNA using polymerase chain reaction (PCR) techniques.

## Neurologic deficit and outcome of surgery

In the MRC trials, patients with paraparesis “severe enough to prevent walking across a room” were not eligible for randomization [6]. These patients with significant neurologic deficit clearly required surgical decompression.

But there is evidence to suggest that those with stable, non-progressive and partial neurologic deficit can recover with conservative treatment alone [6, 7, 14–16]. In Tuli’s paper [17] in 1969, he reported about 50 % of 100 consecutive patients that made a good recovery from paraplegia with bed rest and anti-tuberculous therapy alone. Patients were kept on best rest for 9–12 months followed by bracing for another 18–30 months. Surgery would be considered if there were further deterioration in partial neurologic deficit (though the degree of paraparesis was not clearly defined), or if there was lack of improvement after 3–4 weeks of bed rest and chemotherapy. From this, he developed the idea of the ‘middle path regimen,’ which has been proposed for places with lesser resources [14, 15].

In the two Korean centers all the patients were treated conservatively. They were either prescribed in-patient bed rest, ambulatory chemotherapy with plaster jacket or out-patient chemotherapy. Twenty-eight out of 283 patients presented with paraparesis, and 5 more developed paraparesis while on treatment. Though overall there was 88 % favorable outcome at 10 years, a careful look at those patients with partial neurologic deficit revealed 11 out of 33 (33 %) had rather poor outcomes [8]. Two died paraplegic despite salvage surgery, and the remaining nine had relapsed or prolonged chemotherapy requirements. These patients often suffered from residual spasticity and walking disturbance.

South Africa had an even higher percentage of patients with deficit—27 % were paraplegic and 12 % were paraparetic [5]. At 3 years, around two-thirds of those recovered the functional use of the lower limbs. Though it should be emphasized that this does not equate to having full recovery.

Radical debridement and strut grafting appeared to have an advantage over the above approaches with over 80 % attaining good recovery. Bailey et al. [16] gave an account of the first 100 consecutive children (10 years or less by age) treated by radical anterior debridement and strut grafting in Hong Kong, and followed up for 8 years. Nineteen out of 23 (83 %) of those with “incomplete paraplegia”, defined as those with evidence of cord compression but still able to walk, had complete recovery. In the remaining four that had partial recovery, three patients had lower limb hyper-reflexia and one had weakness requiring a brace. For those with “complete paraplegia”, defined as those unable to walk without support, 17 out of 20 (85 %) recovered completely. Those in the complete paraplegia group took longer before they sought medical help, and on average waited 15.5 months. For neurologic recovery to plateau it may take as short as 6 months, but could be as long as 36.

Furthermore, conservative treatment of patients with neurologic deficit, while successful, may take more time for the neurology to resolve and the patient to become ambulant. With improvements in healthcare, and increase in expectations from the patients and society, it would be reasonable to consider early surgical decompression to allow more rapid resolution of neurologic deficit, and an earlier return to work and an active lifestyle. Thus, many recent reports have described various procedures to decompress and stabilize the spine to allow early mobilization (see below).

## What constitutes significant instability or deformity?

With the destruction of the anterior column of the spine by an infective focus, kyphosis may result from the progressive anterior collapse. When surgery is indicated for the prevention or treatment of kyphosis, a number of points are worth considering:

1. How much residual kyphosis is acceptable?
2. Can children be treated more conservatively due to their ability to remodel?
3. What is the best surgical option to prevent progression of kyphosis?

Despite these well documented problems and a number of long-term follow up reports on post-TB kyphosis, there appears to be *no detailed study* on the degree of kyphosis that will lead to late onset paraplegia or cardiopulmonary compromise. A commonly subscribed figure has been 60°, and this figure has been given by Rajasekaran, Jain and Tuli [18–20]. Surgical treatment should be rendered if there were 60° kyphosis since it appeared to cause the most morbidities.

**Fig. 2:**
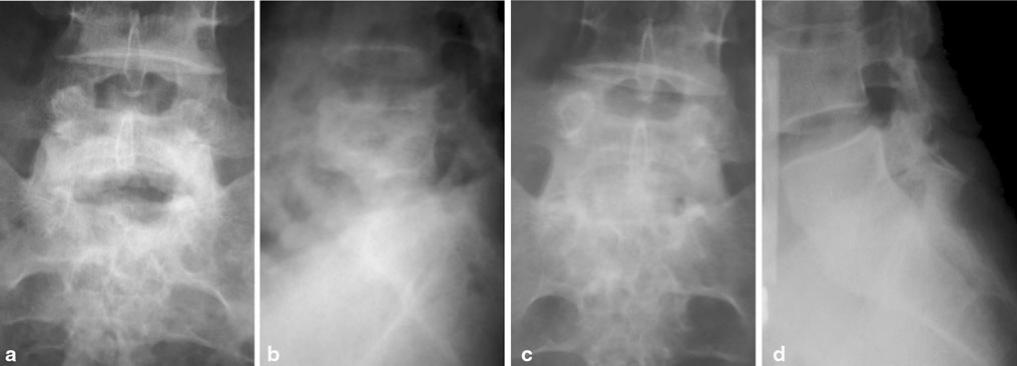
Illustration of the preservation of lordosis and maintenance of disk height at the lumbosacral junction using the wider iliac crest bone graft, when comparing early post-operative AP (**a**) and lateral (**b**) films to that of consolidated fusion site (**c**, **d**)

What would seem sensible is that more kyphosis could be accepted in the thoracic spine, where a compensatory lordosis in the lumbar spine may be able to maintain a satisfactory sagittal balance. But in the lumbar spine, a loss of lordosis would not be so well tolerated, especially if this occurs at the lumbosacral junction. A review from Hong Kong showed that conservative treatment in this area resulted in higher incidence of back pain and kyphosis, when compared to radical surgery and strut fusion (Fig. 2) [21].

Hsu [22] reviewed 22 patients with late-onset paraplegia and found that the kyphotic angle varied from 80° to 160°, and age varied from 12 to 38 years. Thus, suggesting that angulation less than 80° may not cause significant neurologic compromise. These patients presented on average 18 years (from 5 to 33 years) after first diagnosed with TB spine. While in adults, a healed kyphosis may not progress, as will be discussed below, children may experience progression with time, and thus, the kyphotic angle that started with the gradual deterioration may not be as high as 80°.

There are only a few large-scale, long-term studies on the natural history of *healed* *kyphosis* *in adults*. In most studies, including the MRC trials, subjects were predominantly children or at least patients less than 16 years of age. The exception was Hong Kong where over 40 % of study population was 20 years or older [12]. No subgroup analysis of the different age groups were available but it can be appreciated that there was no increase in kyphosis in the 15-year follow up among those that had the Hong Kong operation, which produced a surer and faster union.

For children, Rajasekaran [23] proposed calculating the “instability score” to determine the likelihood of progression. This was an attempt to quantify the degree to which the posterior arch has broken down, based on assessment of lateral X-rays, and thus predict whether continued deterioration in kyphosis will occur. It is calculated by adding 1 point for each specific radiologic “*spine at risk*” sign separation of facet joints, posterior retropulsion of diseased vertebrae, toppling sign, and on AP view lateral translation of one vertebra on another. If greater than 2, he asserted that it allowed one to “accurately predict an increase in the angles of deformity and kyphosis of more than 30° and a final deformity or more than 60°.” The author himself admitted that once facet dislocation was noted, the other signs usually came about quickly; there were only 3 patients out of 63 scoring between 0 and 3. Also of note was that those older than 10 years of age essentially followed an adult pattern of progression.

It was apparent that the progression of kyphosis is mainly determined by how acute and how severe the curve is, with posterior arch integrity playing a critical role in the *tension band effect* that prevents further “*buckling*” in the spine [24]. While growth in children may be a cause for worry of deterioration, it may also allow decrease in kyphosis especially in cases that were operated using the Hong Kong operation [9, 12]. The faster bony fusion attained using this procedure, and thus healing of the predominantly anterior disease, may be especially important in the growing spine.

In short, there is a general consensus that 60° kyphosis is significant and should be addressed surgically. In children, due to the growth potential, there may still be changes in the kyphotic angle after healing, and those cases with a “spinal instability score” of greater than 2 should be tackled early. For adults a solid fusion may preclude deterioration, though a large kyphus may have other medical and psychosocial implications, including Pott’s paraplegia of late onset.

## What constitutes failure of conservative management?

Surgery is also indicated, when there is a failure of conservative treatment, either for pain or neurologic deficit. Pain may arise from persistent infection, progressive destruction of vertebral structure, or the instability and deformity that ensue. The latter may be painful itself but may also cause nerve root impingement. Failure is construed when there is a lack of response to chemotherapy, with or without brace or bed rest, after a reasonable period, usually taken as 3–4 weeks.

Large abscesses may also cause pain, but modern imaging equipment has made possible procedures such as CT-guided drainage. Adequate drainage could often be obtained using these percutaneous techniques at least for iliopsoas abscesses [25]. Anterior cervical and thoracic abscesses may prove to be too difficult. And as the clearance of abscess plays a vital role, surgical drainage will be necessary in these cases.

## Uncertain diagnosis may necessitate surgical biopsy

The discussion on diagnosis is dealt with in another article. Though TB may be rare in places that it is not endemic [26], the index of suspicion should not be lowered least the diagnosis be delayed. If there were compatible history and radiologic findings supported by an extra-spinal culture sample yielding *Mycobacterium tuberculosis*, then the circumstantial evidence may allow us to make the diagnosis of acute TB spondylitis [27]. On the other hand, when there is no easy target from a discharging sinus or a superficial abscess, or the specimen obtained by image-guided percutaneous biopsy was insufficient, then the only means to obtain a specific culture and sensitivity report is by way of open surgical biopsy. Even with PCR studies, which have enhanced the speed and sensitivity with which we diagnose TB, the need for an antibiotic sensitivity study may warrant the open biopsy [28].

In recent years, interferon-gamma assays have been proposed as an equivalence test to tuberculin skin test, especially in its use for diagnosing latent TB [29]. It may prove useful in the setting of typical radiologic signs but an absence of specific culture results, though further studies are still ongoing with regards to its application and limitations.

## Which region of the spine is radical debridement and strut graft indicated and how much better is it?

It is evident from the introduction and the discussion on neurologic indications for acute surgery that the Hong Kong operation offers two advantages, which were not initially appreciated in the MRC reports, namely faster resolution with less long-term kyphosis [30]. In addition, there was no relapse or recurrence of sinus and abscess. This truly favorable outcome provides testament to the importance of an anterior approach that would allow “extirpation” of the tuberculous focus [11].

Tuberculosis of the spine is mostly concentrated in the thoracolumbar spine, and the MRC trials excluded patients with *only cervical* lesions. However, for both subaxial cervical spine and the lumbosacral junction, the principles employed in the Hong Kong operation appear to apply.

For cervical spine, the main difference was a much higher percentage of cord compression compared to those involving thoracolumbar spine. In Hong Kong, it was about 43 % on average, and adults had a greater predisposition compared to children [31]. In the Delhi series, it was noted in 61 % of subaxial spine lesions [32], and following the “middle path” regimen and they achieved 90 % recovery rate. This was in contrast to two other series that used the Hong Kong operation and achieved full recovery in all the patients [31, 33]. Cervical lordosis also improved by 25° when the latter surgical technique was employed.

## When is instrumentation indicated?

**Fig. 3:**
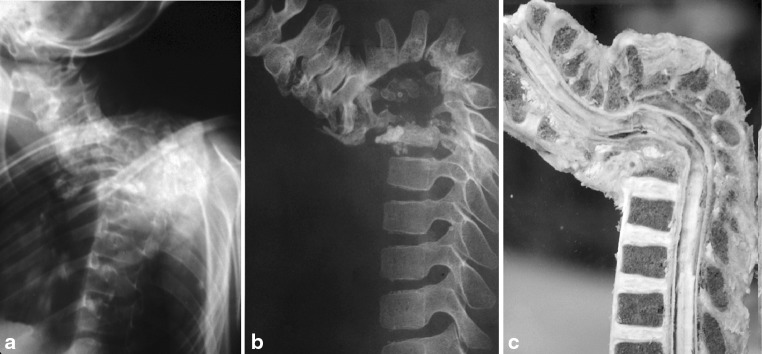
The extent of anterior column deficit that may result from multi-level disease which not be easily appreciated from an X-ray (**a**), but is more evident from CT scan (**b**) and well illustrated in the pathological specimen (**c**) which also shows the extent of posterior ligament stretch

In the Hong Kong operation, patients were kept in plaster beds for an average of 73 days after surgery [3]. Though the end plate bedding for seating the strut graft is prepared till healthy bleeding is seen [3, 16] some subsidence may still be expected, and Hodgson's group already commented on how it may be advantageous to instrument or fuse posteriorly if more than two vertebrae were involved (Fig. 3). In a Madras study (jointly sponsored by the MRC Working Party on Tuberculosis of the Spine) failure of the strut graft was shown to be dependent on the number of segments destroyed [34]. The authors concluded that *graft length spanning 2 disk spaces* should have supplemental measures to prevent collapse.

Posterior fusion and instrumentation obviates the need for bed rest and prolonged bracing. Furthermore, it affords the opportunity for further correction of an exaggerated kyphosis. Around 10 % of cases have a kyphotic angle greater than 40° at presentation [4, 6]. With more severe kyphosis, there may be a greater extent of posterior arch deficit, and potentially, more soft tissue release may be needed to achieve a given degree of correction. Correction of kyphosis and extensive release both add strain to the graft-host bone interface.

This has prompted some to instrument even short segments [35]. While there is not enough evidence at this stage to conclude how many levels need to be instrumented, Luk [36] reasoned that for short segment diseases instrumenting one level above and below the affected vertebra should suffice. Flamme [37] has shown that for instrumentation spanning no more than two disk spaces, there was no advantage of posterior over anterior with regards to axial and side bending loads. But for multi-level disease, multi-level instrumentation would definitely be necessary to confer adequate stability [38].

There may be worry about instrumenting an infection case, but this had been laid to rest by Oga [39] in their detailed study of posterior instrumentation using stainless steel implants. More recently, *tuberculosis* has been shown to have more favorable adherence properties than *Staphylococcus*, and this characteristic was demonstrated in titanium implants as well [40, 41].

**Fig. 4:**
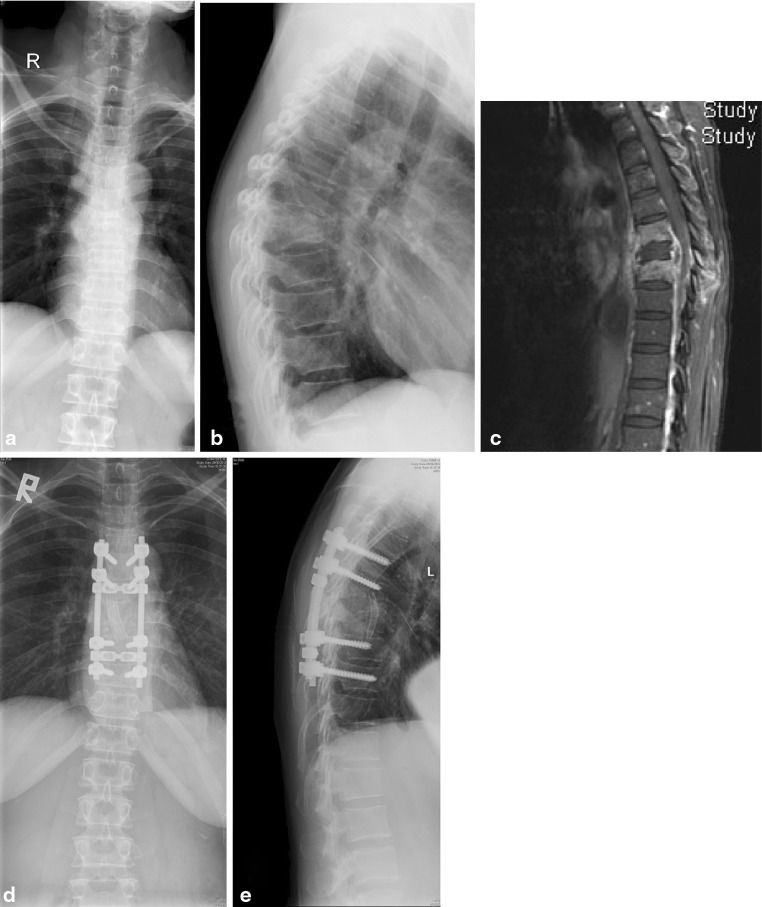
A case requiring anterior debridement, and rib strut grafting (the “Hong Kong Operation”) combined with posterior instrumentation to preserve the sagittal alignment in a case with multi-level infection. Pre-operative X-rays (**a**, **b**) and the MRI (**c**) showing the extent of involvement, and post-operative X-rays (**d**, **e**) showing restoration of normal kyphosis

Thus either anterior or posterior instrumentation may be considered without significant untoward increase in persistence of TB infection. And it is recommended in cases that require radical debridement *spanning two disks and a vertebral body* (Fig. 4). Short segment afflictions may be apt for anterior instrumentation, but for multi-level disease multi-level posterior instrumentation should be considered.

## When is a posterior or a combined anterior- and posterior-approach indicated?

It is clear that an *anterior* approach affords the most logical and direct means of addressing a TB spine lesion, which predominantly affects anterior elements. But for a patient with pan-vertebral disease, or with the need for posterior column shortening to reduce the kyphosis, or multi-level disease, *posterior* *stabilization* is necessary.

With the prospect of posterior instrumentation, different approaches have been developed. Earlier series used two-stage procedures to achieve these goals [42, 43]. Subsequently, single-stage procedures were proposed to circumvent the need for turning the patient, thus reducing operative time and repositioning of a less than stable spine. Jain [44] proposed an extrapleural anterolateral approach to allow both anterior and posterior aspects of the procedure to be dealt with simultaneously.

And more recently, Zhang et al. [45] reported a case series of multi-level non-contiguous thoracic TB employing a posterior transforaminal approach to perform an anterior debridement, interbody and posterior fusion and posterior instrumentation. The risk of incomplete debridement and spinal cord injury, however, does not lend the procedure to wide scale use.

In the end, technical expertise and preference of the surgeon(s) should dictate the approach. The posterior approach alone is rarely indicated, maybe except in cases when there is isolated posterior disease or in cases with multi-level non-contiguous involvement. In these cases, a laminectomy is indicated, but should always be accompanied by a fusion and/or instrumentation to prevent development of kyphosis [46].

## Conclusion

Spinal tuberculosis mostly involves the anterior elements over the thoracolumbar segments. Though anti-tuberculous therapy is the mainstay of treatment, neurologic deficit, instability and deformity (either progressive deformity in the acute setting, or the predicted deformity) may compromise the vertebral column and/or spinal cord. Radical debridement and strut grafting (the Hong Kong operation) forms the basis for surgical intervention, and with supplemental instrumentation the results have been even more promising. Subaxial cervical and lumbosacral involvement are less common but the same principles apply, with similarly good results.
